# Corrosion Behavior of Steel-Reinforced Green Concrete Containing Recycled Coarse Aggregate Additions in Sulfate Media

**DOI:** 10.3390/ma13194345

**Published:** 2020-09-29

**Authors:** Abigail Landa-Sánchez, Juan Bosch, Miguel Angel Baltazar-Zamora, René Croche, Laura Landa-Ruiz, Griselda Santiago-Hurtado, Victor M. Moreno-Landeros, Javier Olguín-Coca, Luis López-Léon, José M. Bastidas, José M. Mendoza-Rangel, Jacob Ress, David. M. Bastidas

**Affiliations:** 1 Facultad de Ingeniería Mecánica y Eléctrica, Doctorado en Ingeniería, Universidad Veracruzana, Xalapa 91000, Veracruz, Mexico; liagiba07@hotmail.com; 2 National Center for Education and Research on Corrosion and Materials Performance, NCERCAMP-UA, Department of Chemical, Biomolecular, and Corrosion Engineering, The University of Akron, 302 E Buchtel Ave, Akron, OH 44325, USA; jb394@zips.uakron.edu (J.B.); jtr45@zips.uakron.edu (J.R.); 3 Facultad de Ingeniería Civil—Xalapa, Universidad Veracruzana, Lomas del Estadio S/N, Zona Universitaria, Xalapa 91000, Veracruz, Mexico; lalanda@uv.mx; 4 Facultad de Ingeniería Mecánica y Eléctrica, Universidad Veracruzana, Xalapa 91000, Veracruz, Mexico; rcroche@uv.mx; 5 Facultad de Ingeniería Civil—Unidad Torreón, UADEC, Torreón 27276, Mexico; grey.shg@gmail.com (G.S.-H.); vmmorlan@gmail.com (V.M.M.-L.); 6 Grupo de Investigación DICSO, Instituto de Ciencias Básicas e Ingeniería, UAEH, Hidalgo 42082, Mexico; jolguin77@gmail.com (J.O.-C.); luis_lopez@uaeh.edu.mx (L.L.-L.); 7 National Centre for Metallurgical Research (CENIM), CSIC, Ave. Gregorio del Amo 8, 28040 Madrid, Spain; bastidas@cenim.csic.es; 8 Facultad de Ingeniería Civil, Universidad Autónoma de Nuevo León, Ave. Pedro de Alba S/N, Ciudad Universitaria, San Nicolás de los Garza 66455, Mexico

**Keywords:** corrosion, AISI 304 SS, AISI 1018 CS, green concrete, recycled coarse aggregate, sugar cane bagasse ash, Na_2_SO_4_

## Abstract

Novel green concrete (GC) admixtures containing 50% and 100% recycled coarse aggregate (RCA) were manufactured according to the ACI 211.1 standard. The GC samples were reinforced with AISI 1080 carbon steel and AISI 304 stainless steel. Concrete samples were exposed to 3.5 wt.% Na_2_SO_4_ and control (DI-water) solutions. Electrochemical testing was assessed by corrosion potential (*E*_corr_) according to the ASTM C-876-15 standard and a linear polarization resistance (LPR) technique following ASTM G59-14. The compressive strength of the fully substituted GC decreased 51.5% compared to the control sample. Improved corrosion behavior was found for the specimens reinforced with AISI 304 SS; the corrosion current density (*i*_corr_) values of the fully substituted GC were found to be 0.01894 µA/cm^2^ after Day 364, a value associated with negligible corrosion. The 50% RCA specimen shows good corrosion behavior as well as a reduction in environmental impact. Although having lower mechanical properties, a less dense concrete matrix and high permeability, RCA green concrete presents an improved corrosion behavior thus being a promising approach to the higher pollutant conventional aggregates.

## 1. Introduction

Traditionally, the world’s most widely used building material is hydraulic concrete that, when combined with AISI 1018 carbon steel (CS) rebars, forms a system known as reinforced concrete. Reinforced concrete structures are known for their long-lasting service life and low-maintenance requirements. However, due to the corrosion of the steel reinforcement, billions of dollars are spent in the repair and maintenance of bridges, tunnels, roads and docks, among others, by each country [1,2,3,4,5]. The corrosion of steel embedded in concrete is an electrochemical process in which the oxidation of iron occurs at the anode, whereas at the cathode, oxygen reduction takes place. Corrosion occurs due to several factors that promote passivity breakdown, primarily the carbonation or the ingress of aggressive ions [6,7]. The aggressive depassivating ions are chlorides, present in marine environments [8,9,10] and sulfates from inorganic salts normally present in both groundwater and in surface water. However, the concentration of aggressive agents in these environments can be highly variable [11,12,13,14]. The presence of sulfates in contact with a hardened cement paste can significantly increase the solubility of matrix components and cause degradation of concrete through leaching, thus decreasing the degree of protection of the reinforcement [15,16,17]. In other studies, laboratory simulations also show that the galvanized reinforcements outperform traditional carbon steel reinforcements not only in aggressive environments, but also in contact with contaminants found in the concrete mixture [18,19,20,21].

Presently, the use of ordinary Portland cement (OPC) is responsible for 10% of global CO_2_ emissions, a value that can increase up to 15% in the near future [22]. As a solution to this highly pollutive binder, different approaches combining reduced greenhouse emissions and acceptable corrosion resistance properties have been proposed, such as new alkali-activated materials. Some examples of these novel binders are fly ash (FA), slags, metakaolin sugar cane bagasse ash (SCBA) or rice husks ashes (RHA), among others [19,20]. Interest in SCBA and RHA has recently increased due to the fact that both are an agricultural waste product with a similar corrosion performance to OPC [23,24]. After being treated, the SCBA shows pozzolanic activity, making it a suitable binder to replace OPC [24]. However, the required post-treatment to obtain the binder can increase the greenhouse emissions or decrease the workability of the concrete, apart from the mechanical and chemical properties as presented by Franco-Luján et al. [25]. Regarding corrosion behavior, few studies can be found considering these novel binders. For instance, FA in some studies presents a lower diffusion coefficient than OPC [26,27]. Although SCBA presents lower workability, substitution of OPC ranging between 10% and 30% reduces not only the diffusion coefficient of chloride ions, but also the permeability [25,28,29,30,31,32]. As a result, their use has been limited to supplementary cementitious materials (SCMs) as a conservative solution due to the lack of agreement on their corrosion performance [26,27,28,29,30,31,32,33,34,35,36,37,38]. This partial replacement of the OPC presents an environmentally friendly and cost-effective approach due to the by-product’s nature of the novel binders [39,40,41,42].

Furthermore, the recycling of concrete is considered a key process in the current sustainable development trends. This is because concrete is widely used as a construction material. Its manufacturing consumes a large amount of nonrenewable natural resources: aggregates (80%), OPC (10%), SCM (3%) and water (7%). The natural aggregates (NA) used in the manufacturing of concrete are inert granular materials such as sand, gravel, or crushed stone. Gravel and natural sand are generally obtained from a well, river, lake, or seabed [43]. Currently, the global production of aggregates is estimated to be 40 trillion tons, which leads to the exhaustion of natural resources, high energy consumption and extreme impacts on the environment [44].

For the aforementioned reasons, recycled coarse aggregate (RCA) as a replacement for natural coarse aggregate (NCA), in addition to replacing OPC by 20% with SCBA, represents a substantial reduction in the environmental impact of concrete manufacturing [44]. This topic is of great concern in Europe and in developed countries such as the USA and Canada, among others [45]. A total of 78,000 tons of RCA were used in the Netherlands in 1994, due to the fact that the use of 20% RCA thick did not differentiate properties of fresh or hardened concrete, according to the corresponding national organization [46]. The increasing trend of research efforts of RCA for the manufacturing of new concrete has also increased the interest in the production of high-performance, high-strength concrete [47]. It should be noted that the use of thick RCA (up to 30%) is usually recommended, but it is often considered necessary to add superplasticizers [48] to achieve the required workability of the new concrete. These materials can improve the durability of concrete [44,45,46,47,48,49,50,51,52,53,54]. Due to the scarce works found in the literature, further research efforts are needed to determine the effect of the RCA as well as the partial substitution of OPC with SCBA in the corrosion performance of these novel concretes [55,56,57].

The aim of this work was to study the effect of the substitution of NCA by the environmentally friendly RCA on the GC embedding AISI 1018 carbon steel (CS) and AISI 304 SS rebars. This GC was also partially substituted with SCBA to further decrease the environmental impact of the traditional OPC concrete. Furthermore, the mechanical strength of the new GC was investigated to describe its future real-world applications. Five different concrete mixtures were prepared according to the ACI 211.1 standard [58], two reinforcement alloys, AISI 304 SS and carbon steel 1018, were investigated under control and aggressive environments. Corrosion monitoring techniques, such as open circuit potential (OCP) and linear polarization resistance (LPR), were used to elucidate the corrosion behavior of the novel green concretes. This work contributes to the corrosion performance knowledge as there is not a clear mechanism on how RCA affects the corrosion phenomenon. Furthermore, it presents concrete mixtures with a substantial reduction in the environmental impact due to the partial substitution not only of OPC with SCBA, but also the natural aggregates by the RCA, thus reducing the CO_2_ emissions substantially [22].

## 2. Materials and Methods

### 2.1. Green Concrete (GC)

**Figure 1:**
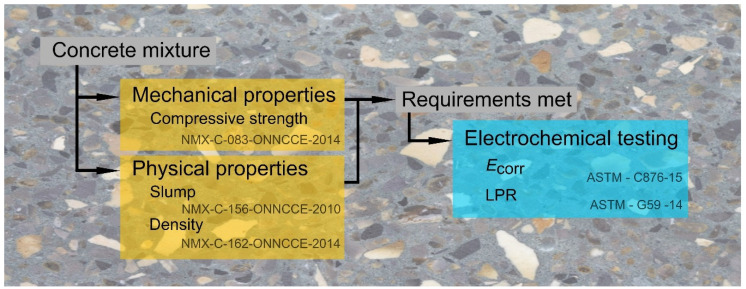
Experimental testing procedure schematic.

Three different concrete mixtures were made: a conventional concrete control mixture (MC) made with 100% OPC following the standard for Portland blended cement (CPC 30R, NMX-C-414-ONNCCE-2014) [59], natural fine (NFA) and coarse (NCA) aggregates and two mixtures of green concrete (GC)—the first green concrete with a 50% substitution of NCA for RCA and with a partial 20% substitution of cement for SCBA, and the second green concrete with a 100% substitution of RCA and the same SCBA ratio. The SCBA was obtained from Mahuixtlan sugar mills, located in Coatepec, Mexico. The characterization of the physical properties of aggregates, NCA, NFA and RCA, was made in accordance with the ASTM standards, the tests were relative density (specific gravity) [60,61], bulk density (unit weight, kg/m^3^) [62], absorption (%) of coarse aggregate and fine aggregate [63], maximum aggregate size and fineness modulus [58]. Figure 1 shows the proposed experimental testing procedure to determine the optimal mixture design. Table 1 shows the physical properties of the materials in this research.

### 2.2. Design Mixtures of Conventional Concrete (MC) and GC

The design of concrete mixtures for MC and GC created according to the standard ACI 211.1 [58]. This standard describes a method that is based on the physical properties of coarse and fine aggregates (see Table 1). The proportioning of the concrete mixture indicates the amount of material needed to produce a meter cubic of concrete. In this case, the manufacture of the three concrete mixes used a water/cement ratio of 0.65 for a specified compressive strength of concrete ($f_{c}^{\prime }$ = 22.5 MPa according to ACI 214R-11 [64]). Table 2 summarizes the proportions for the MC and the two GC mixtures (M50 and M100).

### 2.3. Physical and Mechanical Properties of Concrete Mixtures (Fresh and Hardened State)

For the evaluation of the physical properties of fresh-state concrete mixtures, tests of slump [65], freshly mixed concrete temperature [66] and density [67] were carried out according to the ONNCCE and ASTM standards. Table 3 shows the results obtained for the two concrete mixtures.

To determine the mechanical strength (compressive strength, $f_{c}^{\prime }$) of the concrete mixtures in the hardened state, compression tests were carried out according to the standard NMX-C-083-ONNCCE-2014 [68], at the ages of 14 and 28 days. Table 4 shows the results obtained.

The compressive strength decreased as the content of recycled coarse aggregate (RCA) present in GC increased. The GC mix with 50% RCA and 20% SCBA was substituted for the cement CPC 30R (M50) and showed a compressive strength of 11.54 MPa at 28 days. This represents a decrease of 42% with respect to the MC, and a decrease of 51.5% for GC with 100% RCA and 20% SCBA replacing cement CPC 30R, reporting a compressive strength of only 9.66 MPa at an age of 28 days. The decrease in compressive strength in GC mixes is related to the incorporation of RCA. This behavior agrees with that reported in various investigations. Ali et al. found in their investigation of glass fibers incorporated in concrete with RCA that when RCA completely replaces NCA, it reduces the compressive strength, split tensile strength and flexure strength by about 12%, 11% and 8%, respectively [69]. Kurda et al. concluded that both materials, FA and RCA, are detrimental to the mechanical properties of concrete. For instance, compressive strength, splitting tensile strength and modulus of elasticity are negatively affected. The SiO_2_ present in the FA and the Ca(OH)_2_ present in the RCA experience a pozzolanic reaction that increases the rate of concrete strength development over time [70]. The SiO_2_ is also present in the SCBA according to previous results [71], thus being a likely source of this detrimental behavior. Li et al. explained in their research in the structural area that there is a reasonable consensus regarding the structural behavior of composite members combined with RCA. Mechanical strength is slightly lower compared with OPC with no RCA additions. Nevertheless, the manufacturing of composite materials using RCA presents a safe and feasible approach [72]. However, the compressive strength observed for GC was sufficient for use in structures that do not require high strength, such as houses, parks, sidewalks, floors, etc.

### 2.4. Specifications, Characteristic and Nomenclature of Specimens for Electrochemical Tests

The MC and the two mixtures of GC (M50 and M100) were made with a water/cement ratio of 0.65. The specimens were prisms with dimensions of 15 × 15 × 15 cm. In all the specimens, AISI 304 SS and AISI 1018 CS rebars were embedded with a length of 15 cm and a diameter of 9.5 mm; the AISI 304 SS and AISI 1018 CS rebars were cleaned to remove any impurities [73]. In addition, each rebar was coated 4 cm from the top and 4 cm from the bottom using insulating tape in order to limit the exposed area with a length of 5 cm, as reported previously [74,75].

**Figure 2:**
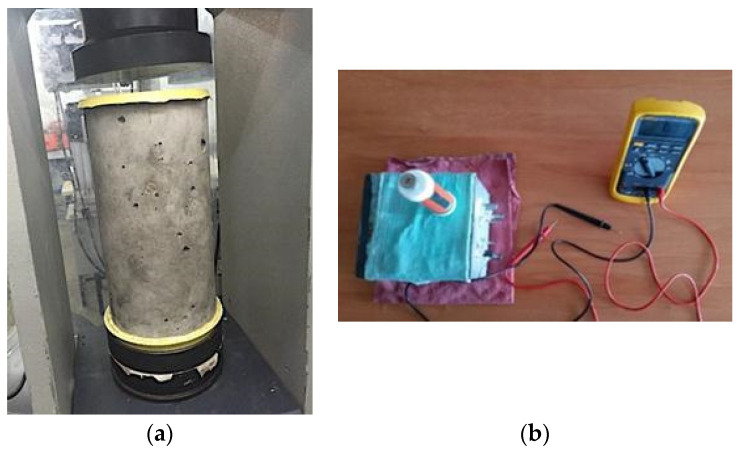
Experimental test conducted on green concrete: (**a**) compressive strength and (**b**) electrochemical corrosion monitoring.

The specimens were manufactured in accordance with the standard ASTM C 192 [76] and the curing stage of all specimens was carried out water immersion according to the NMX-C-159 standard [77]. After the curing period, the eight specimens were placed in the exposure media, a control medium (DI-water) and 3.5 wt.% Na_2_SO_4_ solution for 364 days, simulating a sulfate aggressive medium such as contaminated soils, marine and industrial environments [78,79]. The specimens were then subjected to electrochemical tests. Figure 2 shows the compressive strength tests of the different GC mixtures and the electrochemical test to determine the corrosion behavior after exposure to 3.5 wt.% Na_2_SO_4_ solution.

Table 5 shows the elemental composition of the austenitic AISI 304 stainless steel and AISI 1018 carbon steel.

The nomenclature used for the electrochemical monitoring of AISI 304 SS and AISI 1018 CS embedded in the MC and the two GC (M50 and M100) exposed in a control medium (DI-water) and 3.5 wt.% Na_2_SO_4_ solution is shown in Table 6, which has the following meaning:

- MC, M50 and M100 indicate the concrete mixture (conventional and green concrete);
- W indicates exposed DI-water (control medium);
- S indicate exposed to 3.5 wt.% Na_2_SO_4_ solution (aggressive medium);
- 18 for rebars of AISI 1018 CS;
- 304 for rebars of AISI 304 SS.

**Figure 3:**
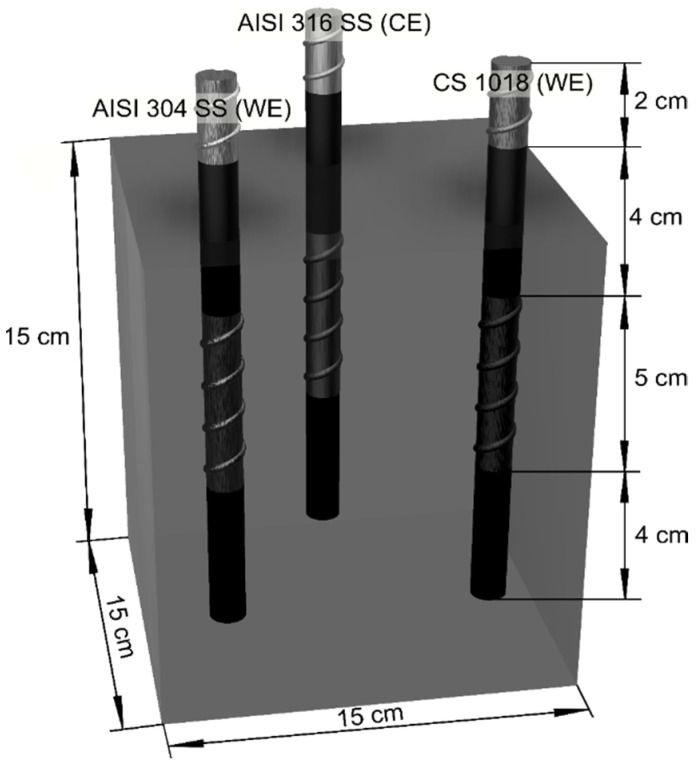
Specifications of specimens for electrochemical tests.

MC and GC specimens were exposed to two different media, the control medium (DI-water) and 3.5 wt.% Na_2_SO_4_ solution, for a period of 364 days. The corrosion behavior was characterized by corrosion potential (*E*_corr_) and corrosion current density (*i*_corr_) measurements. The electrochemical cell setup used was AISI 304 SS or AISI 1018 CS rebars with a diameter of 9.5 mm for working electrodes (WE). AISI 314 SS rebars were used as counter electrodes (CE; see Figure 3) and standard copper–copper sulfate (Cu/CuSO₄, CSE) as the reference electrode (RE). *i*_corr_ was monitored using the linear polarization resistance (LPR) technique. The sweep potential range was ±20 mV with respect to the *E*_corr_ and the sweep rate was 10 mV/min according to standard ASTM-G59 [80]. Electrochemical measurements were performed in a Gill AC Galvanostat/Potentiostat/ZRA (ACM Instruments, Cark in Cartmel, UK). The results were analyzed using Version 4 Analysis specialized software from ACM Instruments [81,82]. All tests were carried out at room temperature. *E*_corr_ and *i*_corr_ were monitored every four weeks and all experimental measurements were performed in triplicate.

The *i*_corr_ and the corrosion rate (*v*_corr_) were estimated from the LPR technique using the Stern and Geary relation (see Equation (1)) [83]:

(1) $$i_{\mathrm{corr}}=\frac{B}{R_{p}}$$

where *B* is the proportionality constant equal to 26 and 52 mV/dec for active and passive corrosion state rebars, respectively, and *R*_p_ is the polarization resistance [84,85].

*E*_corr_ was used to assess the degree of deterioration of reinforced concrete specimens according to ASTM C-876-15 [86], which presents the criteria or ranges that relate the *E*_corr_ values with the probability of corrosion for embedded steel specimens made with MC and GC (see Table 7).

To determine the *v*_corr_ values of steels embedded in the mixtures of MC and GC, the *i*_corr_ values were used. The criteria used to analyze the *i*_corr_ results are based on the state of corrosion of CS in OPC reported in the literature [84], as shown in Table 8.

## 3. Results and Discussion

### 3.1. Half-Cell Potential—Corrosion Potential

Half-cell potential monitoring (E_corr_) and interpretation of the corrosion state were performed using the criteria presented in Table 7, which is in accordance with ASTM C876-15 [86].

#### 3.1.1. E_corr_ Specimens Exposed DI-Water (Control Medium)

**Figure 4:**
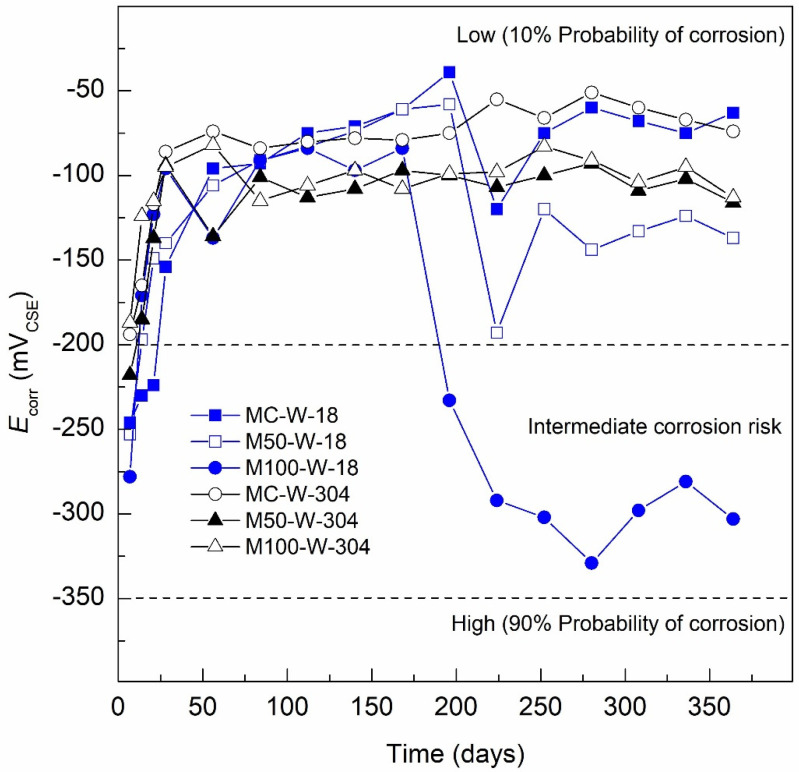
*E*_corr_ specimens exposed DI-water (control medium).

Figure 4 shows the results obtained from monitoring the *E*_corr_, of the specimens MC-W-18, M50-W-18 and M100-W-18. It is observed that the MC-W-18 specimen presents corrosion potentials in the curing stage ranging from −260 to −160 mV_CSE_, moving from Days 7 to 28 from the intermediate corrosion risk to 10% risk, according to ASTM C-876-15. The trend towards more positive values continued throughout the evaluation period, reaching values up to −45 mV_CSE_ on Day 196, and finally reaching values in the range of −60 to −75 mV_CSE_, which indicates a 10% of risk corrosion. For the M50-W-18 specimen, the behavior is very similar to the MC-W-18 specimen, with *E*_corr_ values in the curing stage of −250 mV_CSE_, reaching a value of −140 mV_CSE_ on Day 28, maintaining a trend towards more positive values until Day 196, reaching an *E*_corr_ value of −45 mV_CSE_. At the end of the monitoring period, more negative *E*_corr_ values are found ranging between −120 and −145 mV_CSE_, thus indicating a 10% corrosion risk.

The M100-W-18 specimen shows a more unfavorable behavior from Day 196, from *E*_corr_ values lower than −200 = to −330 mV_CSE_ for Day 280, until values of −300 mV_CSE_ observed at the end of the monitoring period. Therefore, the specimens indicate intermediate corrosion risk according to ASTM C-876-15 and with a tendency to more negative values of *E*_corr_, which agrees with the findings of Al-Yaqout et al.; the corrosion potential for normal mixtures decreases as the RCA replacement level increases compared to the control mixture. This behavior shows the same trend for the compressive strength, decreasing as the amount of RCA increased in all normal and slag mixtures and under all exposure conditions [87].

The MC-W-304 (MC, 100% CPC-100% natural aggregates), M50-W-304 (GC with 50% RCA and 80% CPC-20% SCAB) and M100-W-304 (GC with 100% RCA and 80% CPC-20% SCAB) specimens were reinforced with AISI 304 SS steel rebars. The *E*_corr_ results obtained after more than 360 days of monitoring show the following behavior. The MC-W-304 specimen presented a tendency to more noble *E*_corr_ values from the curing stage, presenting values from −192 mV_CSE_ on Day 7 to −84 mV_CSE_ on Day 28, continuing with a tendency to passive *E*_corr_ values in the range of −80 to −75 mV_CSE_ from Day 196 [88,89], presenting values below or more positive than −200 mV_CSE_, which indicates, according to the ASTM C-876-15, a 10% corrosion risk or passivity of the steel–concrete system analyzed.

The M50-W-304 specimen behaves similarly to the control MC-W-304, with corrosion potentials in the curing stage ranging from −218 mV_CSE_ on Day 7 to −95 mV_CSE_ on Day 28, presenting a small activation on Day 56 with an *E*_corr_ value of −143 mV_CSE_. From this point to the present, a stage of stability in the *E*_corr_ values is observed from Days 84 to 364, in the range of −90 and −120 mV_CSE_, interpreted according to the ASTM C-876-15 as a 10% corrosion risk. The M100-W-304 specimen, presented a similar behavior to the two MC-W-304 and M50-W-304 specimens in the curing stage, showing an *E*_corr_ value of −183 mV_CSE_ on Day 7 and −97 mV_CSE_ on Day 28. From this point on, the *E*_corr_ values become −82 and −124 mV_CSE_ until the end of the testing, indicating according to the ASTM C-876-15 as a 10% corrosion risk. The behavior in the *E*_corr_ values is less than −200 and congruent with the nonaggressive medium of exposure, which is also interpreted as the passivity of the AISI 304 SS steel used as reinforcement in GC and MC.

#### 3.1.2. E_corr_ Specimens Exposed 3.5 wt.% Na_2_SO_4_ Solution (Aggressive Medium)

**Figure 5:**
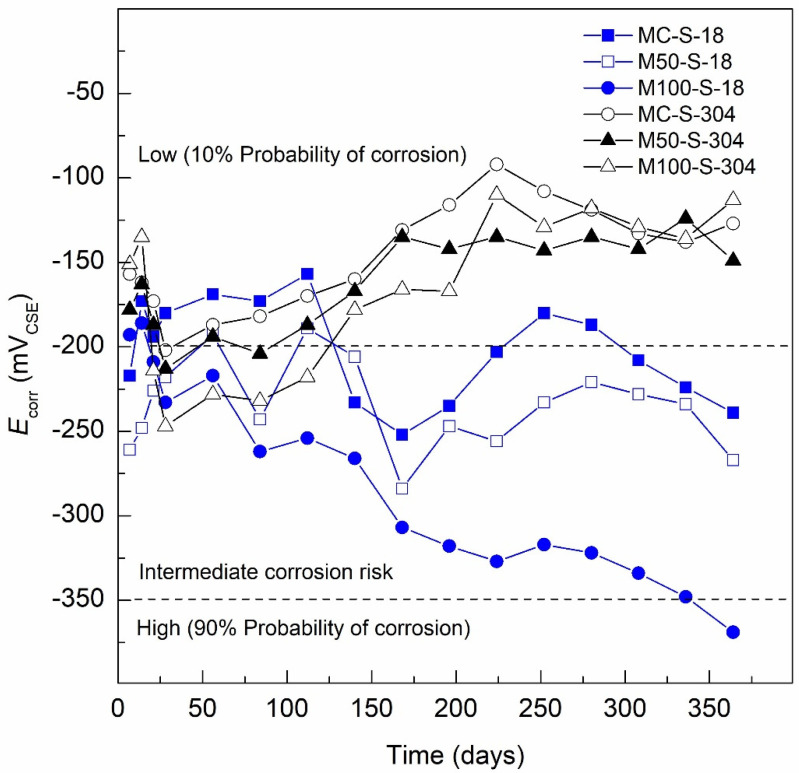
*E*_corr_ specimens exposed 3.5 wt.% Na_2_SO_4_ solution (aggressive medium).

Figure 5 presents the results obtained from the *E*_corr_ monitoring of the specimens when exposed for 364 days to 3.5 wt.% Na_2_SO_4_ solution (aggressive medium). The evaluated specimens were MC-S-18 (MC, 100% CPC-100% natural aggregates), M50-S-18 (GC with 50% RCA and 80% CPC-20% SCAB) and M100-S-18 (GC with 100% RCA and 80% CPC-20% SCAB). The MC-S-18 specimen in the curing stage presented an *E*_corr_ value of −217 mV_CSE_ on Day 7 and −180 mV_CSE_ for Day 28. These *E*_corr_ values indicate, according to the ASTM C-876-15, a 10% corrosion risk. Later, the specimen presents *E*_corr_ values in the range from −173 to −159 mV_CSE_ after Day 112, from this point to the present, an activation occurs with *E*_corr_ values from −203 to −256 mV_CSE_ from Day 140 to 224, which would indicate intermediate corrosion risk according to ASTM C-876-15. For Days 252 and 280, *E*_corr_ values are lower than −200 mV_CSE_, which would be associated with a passivity stage or a 10% corrosion risk; however, after Day 280, there is a trend towards more negative values of −200 mV_CSE_, reaching −239 mV_CSE_ on the last day of monitoring. The M50-S-18 specimen presents more negative values of *E*_corr_ in the curing stage than those presented by the control MC-S-18 specimen, with an *E*_corr_ value of −261 mV_CSE_ on Day 7 and −218 mV_CSE_ for Day 28, showing from Days 56 to 140 *E*_corr_ values that ranged between −189 and −243 mV_CSE_. Then, the specimen shows a decreasing trend towards lower values until the end of the testing, with values reaching −284 mV_CSE_, after Day 140 and until the end of monitoring, Day 364, the *E*_corr_ values for the M50-S-18 specimen when exposed in 3.5 wt.% Na_2_SO_4_ solution (aggressive medium) indicate intermediate corrosion risk according to the ASTM C-876-15.

The specimen that presented the worst performance when exposed to 3.5 wt.% Na_2_SO_4_ solution (aggressive medium) was M100-S-18, presenting a tendency to lower *E*_corr_ values with an *E*_corr_ value of −193 mV_CSE_ on Day 7 of the curing stage and −233 mV_CSE_ for Day 28, continuing with the negative trend throughout the entire exposure period, reaching a potential of −348 mV_CSE_ on Day 336 and ending on Day 364 with a corrosion potential of −369 mV_CSE_. This indicates a <90% corrosion risk according to the ASTM C-876-15 standard. This behavior of more negative corrosion potentials (*E*_corr_) coincides with that reported in other investigations when evaluating AISI 1018 steel in sustainable concrete made with SCBA and exposed to sulfates [24]. However, the M100-S-18 specimen presents more negative values, which is associated with lower corrosion resistance of the specimens made with GC (M50-S-18 and M100-S-18) when exposed to sulfates, related to a less dense matrix and higher permeability due to the presence of 50% and 100% RCA, as well as the substitution of Portland cement in 20% by SCBA. This decrease in the mechanical properties and durability when RCA has been used was reported by Lovato et al. [90], indicating that the durability properties are also negatively affected by the increment of RCA in concrete. In order to achieve the required workability, the water-to-cement ratio must be increased. This not only leads to an increased demand for water during the manufacturing stage, but also an increase in the porosity of the matrix and consequently reducing the durability of the concretes [90].

The specimens with AISI 304 SS steel were MC-S-304 (MC, 100% CPC-100% natural aggregates), M50-S-304 (GC with 50% RCA and 80% CPC-20% SCAB) and M100-S-304 (GC with 100% RCA and 80% CPC-20% SCAB), exposed for 364 days to 3.5 wt.% Na_2_SO_4_ solution (aggressive medium). The MC-S-304 specimen presented an *E*_corr_ value of −157 mV_CSE_ on Day 7 of the curing stage and −202 mV_CSE_ for Day 28, from this point, the specimen presents a trend towards higher *E*_corr_ values, related to the passivity of AISI 304 SS steel, and reached a minimum *E*_corr_ of −92 mV_CSE_ on Day 224 of exposure. Then, the specimen showed *E*_corr_ values in the range from −108 to −138 mV_CSE_ until the end of the monitoring period, all the *E*_corr_ values of the MC-S-304 specimen during the entire period of exposure to the aggressive medium were less than −200 mV_CSE_, thus indicating a 10% corrosion risk according to the ASTM C-876-15. The M50-S-304 specimen presented a behavior similar to MC-S-304, with corrosion potentials in the curing stage with a decreasing trend. The M50-S-304 specimen displays an *E*_corr_ value of −178 mV_CSE_ on Day 7 and −213 mV_CSE_ for Day 28, then increases and become more passive to −138 mV_CSE_ by Day 168 and remains stable in the range of −135 and −149 mV_CSE_ until the final measurement, maintaining *E*_corr_ values below −200 mV_CSE_ throughout the exposure period, thus indicating, according to ASTM C-876-15, a 10% corrosion risk. Finally, the M100-S-304 specimen presents a similar behavior to the two previous specimens in the curing stage, with corrosion potentials ranging from less to more negative, with an *E*_corr_ value of −151 mV_CSE_ on Day 7 and −247 mV_CSE_ on Day 28. Unlike the MC-S-304 and M50-S-304 specimens, the M100-S-304 specimen presents *E*_corr_ values less than −200 mV_CSE_ until Day 112, which would indicate intermediate corrosion risk according to the ASTM C-876-15. Thereafter, the specimen shows a trend towards higher *E*_corr_ values, reaching an *E*_corr_ value of −110 mV_CSE_ for Day 224 and remaining stable in the range between −136 and −113 mV_CSE_ until the end of the testing. Like the previous specimens, the M100-S-304 specimen presented *E*_corr_ values less than −200 mV_CSE_ during almost the entire exposure time to 3.5 wt.% Na_2_SO_4_ solution (aggressive medium), which indicates a 10% corrosion risk according to ASTM C-876-15. The previous results agree with those reported in the literature, where the excellent corrosion resistance of stainless steel grades AISI 304, AISI 316, etc., has been demonstrated when used as reinforcement in conventional concrete, sustainable concrete, green concrete, and when exposed to aggressive environments such as marine, sulfated and industrial environments [91,92].

### 3.2. Corrosion Current Density, i_corr_

The i_corr_ results of the AISI 304 SS and AISI 1018 CS reinforcement in MC and both GC mixtures (M50 and M100) exposed to control medium (DI-water) and 3.5 wt.% Na_2_SO_4_ solution were interpreted according to the criterion of Table 8.

#### 3.2.1. i_corr_ Specimens Exposed DI-Water (Control Medium)

**Figure 6:**
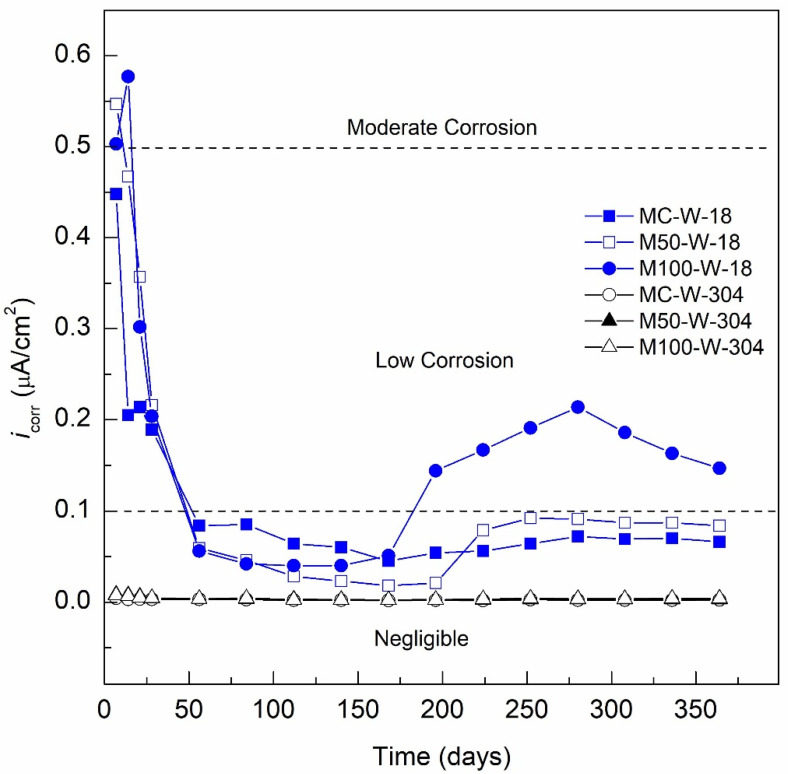
*i*_corr_ specimens exposed DI-water (control medium).

Figure 6 shows the *i*_corr_ results of the conventional concrete and GC specimens reinforced with AISI 1018 CS and AISI 304 SS steel exposed in water as a control medium. The MC-W-18 specimen presents an *i*_corr_ value of 0.67 µA/cm^2^ for Day 7 of the curing stage, decreasing on Day 28 to a value of 0.21 µA/cm^2^. For Day 56, a passive *i*_corr_ value of 0.095 µA/cm^2^ was observed, and subsequently, values remained less than 0.091 µA/cm^2^ until the end of monitoring in the range of 0.09 to 0.05 µA/cm^2^. The *i*_corr_ values obtained from the MC-W-18 specimen indicate passivation of the reinforcing steel and, according to Table 8, a negligible level of corrosion (absence of corrosion). The M50-W-18 specimen presents a similar passivation behavior as MC-W-18; however, with higher *i*_corr_ values from the curing stage, presenting on Day 7 an *i*_corr_ value of 0.58 µA/cm^2^ and 0.29 µA/cm^2^ for Day 28. From Day 56 to the end of monitoring, *i*_corr_ values were below 0.1 µA/cm^2^ in the range of 0.07 to 0.04 µA/cm^2^, indicating a negligible level of corrosion. The M100-W-18 specimen had a similar behavior to the two previous specimens with an *i*_corr_ on Day 7 of 0.64 to 0.26 µA/cm^2^ for Day 28 and presenting an *i*_corr_ value of 0.067 µA/cm^2^ until Day 140. From Day 168 until the end of monitoring, *i*_corr_ values were in the range of 0.144 to 0.214 µA/cm^2^, indicating a low level of corrosion according to Table 8 and supporting the corrosion potential monitoring technique. The corrosion potentials presented by the same M100-W-18 specimen, after Day 168 were in the range of −200 to −340 mV_CSE_, indicating corrosion uncertainty according to ASTM C-876-15. With the LPR test, the *i*_corr_ could be determined, confirming the activation of the system with the presence of a low level of corrosion from Day 196 for the M100-W-18 specimen in a nonaggressive environment. The corrosion present in the M100-W-18 specimen exposed to a nonaggressive medium is related to the less dense and more permeable matrix of green concrete (M100), as indicated by the low compressive strength at 28 days with $f_{c}^{\prime }$ = 9.66 MPa. This decrease in the durability of concrete made with RCA has been demonstrated in various investigations, Kurda et al. found that the water absorption increases and electrical resistivity decreases with the increasing incorporation level of RCA; the opposite occurs with the addition of FA for both tests [93]. The behavior of the *i*_corr_ of the other two specimens, MC-W-18 and M50-W-18, indicated a negligible level of corrosion (passivity).

The MC-W-304 specimen in the curing stage showed an *i*_corr_ value of 0.0043 µA/cm^2^ on Day 7 with a trend towards more passive values, presenting an *i*_corr_ value of 0.0031 µA/cm^2^ on Day 28. A trend to lower *i*_corr_ values is observed until Day 224 with an *i*_corr_ value of 0.0018 µA/cm^2^. Then, the specimen exhibits a small increase of *i*_corr_ to 0.0028 µA/cm^2^ for Day 252 and from *i*_corr_ values of 0.0021 µA/cm^2^ on Day 280 to 0.0023 µA/cm^2^ for the last monitoring on Day 364. All *i*_corr_ values of the MC-W-304 specimen indicate a negligible or null corrosion level according to that indicated in Table 8. It is also found that this specimen presents the lowest *i*_corr_ values, followed by the M50-W-304 specimen, which presented *i*_corr_ values of 0.0085 µA/cm^2^ on Day 7 to 0.0041 µA/cm^2^ for Day 28, then continues with a decrease in *i*_corr_ until Day 168 with a value of 0.0023 µA/cm^2^. Subsequently, the *i*_corr_ increases from 0.0026 to 0.0032 µA/cm^2^ from Days 196 to 364, respectively. Finally, the M100-W-304 specimen (100% RCA and 20% SCBA) presented the highest *i*_corr_ values, presenting an *i*_corr_ value of 0.0045 µA/cm^2^ on Day 28, decreasing to 0.0024 µA/cm^2^ on Day 168. Following, *i*_corr_ increases from 0.0027 µA/cm^2^ on Day 196 to a value of 0.0040 µA/cm^2^ for the last day of monitoring, Day 364. A clear difference is observed in the *i*_corr_ values presented by the three studied specimens, the lowest *i*_corr_ values are shown for the MC-W-304 specimen, followed by the M50-W-304 specimen, and finally the M100-W-304 specimen, the *i*_corr_ range of the three specimens is more than 10 times less than 0.1 µA/cm^2^, which indicates that all the specimens present a negligible level of corrosion throughout the period of exposure to the control medium according to Table 8. The results coincide with what is reported in the literature [21,94,95].

#### 3.2.2. i_corr_ Specimens Exposed 3.5 wt.% Na_2_SO_4_ Solution (Aggressive Medium)

**Figure 7:**
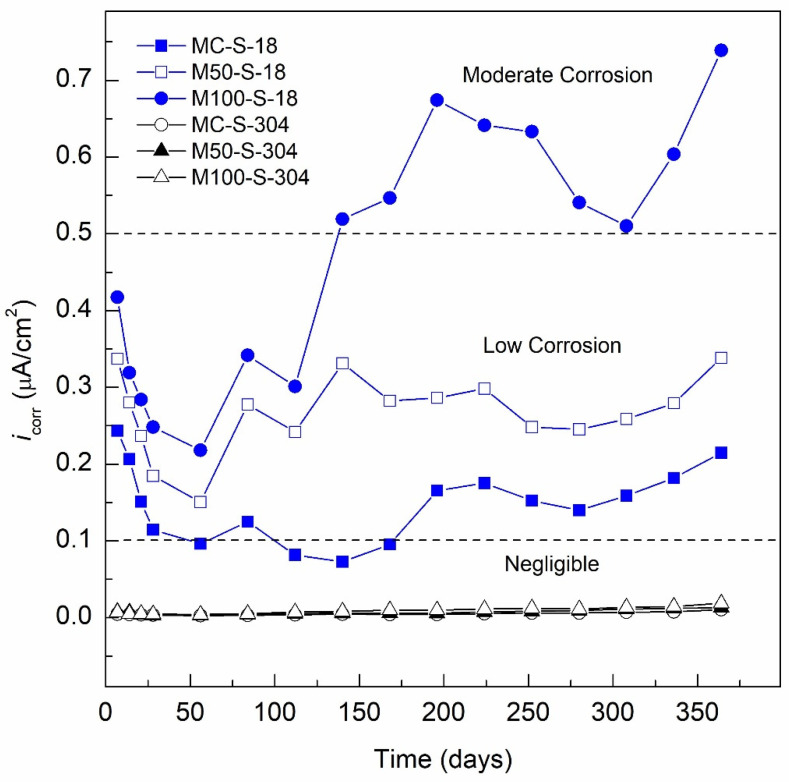
*i*_corr_ specimens exposed 3.5 wt.% Na_2_SO_4_ solution (aggressive medium).

Figure 7 presents the *v*_corr_ and *i*_corr_ results of the specimens with AISI 304 SS and AISI 1018 CS steel bars embedded in MC and GC exposed to 3.5 wt.% Na_2_SO_4_ solution (aggressive medium) for a period of 364 days. The *v*_corr_ and *i*_corr_ of the control specimen, MC-S-18, decreased from an *i*_corr_ value of 0.2435 µA/cm^2^ on Day 7 to an *i*_corr_ value of 0.1144 µA/cm^2^ for Day 28. This behavior is attributed to being in the curing stage where the *i*_corr_ values tend to decrease due to the formation of the passive layer and the increase in the protection of the concrete. The *i*_corr_ values decrease until Day 140 of exposure with a value of 0.0729 µA/cm^2^, indicating a negligible level of corrosion or passivity according to Table 8. However, after Day 168, the activation of the system occurs with a constant increase in *i*_corr_ values greater than 0.1 µA/cm^2^ on Day 196 with an *i*_corr_ value of 0.1656 µA/cm^2^ and reaching 0.2148 µA/cm^2^ at the end of monitoring. This indicates that, as of Day 196, the MC-S-18 specimen presented corrosion at a low level due to the exposure to sodium sulfate solution as an aggressive medium. In the case of the M50-S-18 specimen, the curing stage showed decreasing *i*_corr_ values, reporting 0.3375 µA/cm^2^ on Day 7 to 0.1844 µA/cm^2^ for Day 28. This trend continued to decrease until Day 56, reaching an *i*_corr_ value of 0.1506 µA/cm^2^. However, after Day 84, the *i*_corr_ values begin to increase, becoming more active due to exposure to the aggressive environment and a decreased matrix density and increased permeability because it contains 50% of RCA. The values increase to 0.2779 µA/cm^2^ and remain stable in an *i*_corr_ range of 0.2419 and 0.3386 µA/cm^2^ until the end of monitoring. From Day 84, the M50-S-18 specimen presents *i*_corr_ values that indicate a low level of corrosion according to Table 8. Finally, the M100-S-304 specimen, although showing a tendency for lower *i_corr_* values in the curing stage, displays an *i*_corr_ value of 0.4175 µA/cm^2^ on Day 7 and 0.2482 µA/cm^2^ for Day 28. For Day 86, the activation of the system with an increase in its *i*_corr_ is shown, reaching a value of 0.3417 µA/cm^2^. On Day 140, an *i*_corr_ value of 0.519 µA/cm^2^ indicates a moderate level of corrosion according to Table 8. The *i*_corr_ increases for the M100-S-18 specimen continued irregularly from Day 168 to 308, ending on Day 364 with an *i*_corr_ value of 0.7389 µA/cm^2^. The influence of the 100% RCA in the specimen is observed, influencing the mechanical properties and durability of GC due to a more permeable concrete matrix, lower density and a low resistance to compression compared to the control concrete (concrete with 50 and 100% of coarse natural aggregate). However, the use of mineral admixture (SF, MK, FA and ground granulated blast slag) resulted in a decrease in the charge passed through the concrete specimens [96]. According to Alhawat et al., not only the corrosion initiation process happened faster in RCA concrete, but also a higher corrosion rate was observed as the RCA content increased due to the higher porosity and water absorption [97].

The MC-S-304 specimen presents the best performance against corrosion when exposed for 364 days to 3.5 wt.% Na_2_SO_4_ solution (aggressive medium), reporting *i*_corr_ values in the curing stage of 0.0047 µA/cm^2^ on Day 7 to reach an *i*_corr_ value of 0.0034 µA/cm^2^ on Day 28, observing a decrease associated with the increase in concrete protection due to the hydration process of said stage. The decrease in the corrosion rate occurs until Day 56, when the MC-S-304 specimen reports a minimum *i*_corr_ of 0.0028 µA/cm^2^, from this point, the values stabilize in the range between 0.0039 and 0.0047 µA/cm^2^ between Days 112 and 196 of exposure the aggressive medium. Subsequently, the *i*_corr_ increases gradually from 0.0054 µA/cm^2^ on Day 224 to the highest value in the entire exposure period at the end of monitoring, Day 364, with an *i*_corr_ value of 0.0106 µA/cm^2^. As indicated previously, its performance was excellent in the presence of sodium sulfates, with *i*_corr_ values well below 0.1 µA/cm^2^, which is the limit that would indicate the onset of corrosion according to Table 8. This resistance to corrosion of AISI 304 steel embedded in concrete exposed to aggressive media has been demonstrated in various studies [98,99,100].

In the case of the M50-S-304 specimen, it has a much higher anticorrosive efficiency than that presented by the specimen reinforced with AISI 1018 CS steel (M50-S-18). The M50-S-304 specimen presents *i*_corr_ values in the curing stage ranging from 0.0080 and 0.0031 µA/cm^2^ from Days 7 to 28, respectively. Day 56 shows an *i*_corr_ value of 0.0032 µA/cm^2^, an increase in *i*_corr_ from Day 56 to 196, with constant increases from Days 56 to 112 going from an *i*_corr_ value of 0.0032 and 0.0052 µA/cm^2^, from there to stabilize and oscillate in the range of 0.0058 and 0.0061 µA/cm^2^. From Day 140 to 196, there is a constant increase until the end of the monitoring period, from an *i*_corr_ value of 0.0077 µA/cm^2^ on Day 224 to 0.1321 µA/cm^2^ for the Day 364. Like the MC-S-304 specimen, the *i*_corr_ values are much lower than 0.1 µA/cm^2^, which indicates that its corrosion level is negligible, or passivity occurs, according to the provisions of Table 8. However, it can be observed that the M50-S-304 specimen presents higher values than those reported by the MC-S-304 specimen. This behavior is associated with a less dense and more permeable concrete matrix due to the presence of RCA, as reported by Cakir et al. The compressive strength of the concrete decreases by incorporating RCA and that the presence of RCA causes the concrete to have a higher porosity and lower density [101]. However, another study concluded that the RCA content in the concrete is found to have a detrimental effect in the compressive strength, but at low replacement concentrations <20%, this effect is negligible [102]. The monitored *i*_corr_ values for AISI 304 SS during the curing period were 0.0071 and 0.0047 µA/cm^2^ on Days 7 and 28, respectively, during the curing stage. Next, the *i*_corr_ increases from 0.0041 to 0.0098 µA/cm^2^ for Days 56 to 168, respectively. A second period of increase occurs from Days 196 to 280, from an *i*_corr_ value of 0.00989 to 0.1143 µA/cm^2^. Finally, the third period with near-constant *i*_corr_ of 0.01346 µA/cm^2^ on Day 308 to *i*_corr_ of 0.01894 µA/cm^2^ on Day 364. The *i*_corr_ values during all the periods of exposure showed values less than 0.1 µA/cm^2^, which indicates an excellent performance against sulfate corrosion for the M100-S-304 specimen with 100% of RCA and 20% of SCBA.

The corrosion resistance was not influenced by the high permeability, low density and low mechanical resistance of the GC with which the M100-S-304 specimen was made. By data fitting, the durability properties generally decrease linearly with the increase of RCA replacement and the average water absorption rate [103]. The concrete containing NA and RCA showed a carbonation rate of 1.8 times higher [104]. The increase in the carbonation depth observed in samples containing RCA could be attributed to the higher permeability of RCA due to the presence of old mortar adhering to the NA and the old interfacial transition zone (ITZ) [105]. The geopolymer RCA, with a higher content of granulated blast furnace slag, had a lower mass loss and a higher residual compressive strength after the sulfate exposure [106]. The results indicate a direct influence between the percentage of aggregate used in the GC mixes and the level of corrosion that all the specimens present in both the control medium and the aggressive medium. Higher contents of RCA lead to higher *i*_corr_ in both AISI 1018 CS and AISI 304 SS steels. This behavior is the opposite of the reported behavior in another research, where it was found that the influence on the performance against most usual corrosion processes displayed similar results under a natural chloride attack [107]. Therefore, it is of great importance to continue to study different types of reinforcing steels as an alternative to AISI 1018 steel [108,109] that can increase the resistance to corrosion of GC based on recycled aggregates and alternative materials to OPC, such as SCBA, FA and SF.

## 4. Conclusions

According to the results from the study, the following conclusions were reached:

GC samples showed a significant decrease in the slump in their fresh state, GC-M50 with a slump of 3 cm and GC-M100 with a slump of 2 cm, decreasing their workability compared to conventional concrete (MC) which presented a slump of 10 cm.

The compressive strength shows a decreasing trend as the content of RCA present in GC increases. The GC-M50 mix with 50% RCA and 20% SCBA must be substituted for the CPC 30R. A compressive strength of 11.54 MPa was observed at 28 days, which represents a decrease of 42% with respect to the MC. A decrease of 51.5% for GC with 100% RCA and 20% SCBA replacing CPC 30R. A compressive strength of only 9.66 MPa was seen for Day 28.

The results obtained in the present investigation indicate a direct influence between the percentage of aggregate used in the GC mixes and the level of corrosion that all the specimens present in both the control medium and the aggressive medium, the higher the content of RCA, the higher the corrosion rate in both CS 1018 and AISI 304 SS reinforcements.

The *i*_corr_ values of the GC specimens reinforced with AISI 304 SS exposed to Na_2_SO_4_ were found to be 0.01894 µA/cm^2^ on Day 364, two orders of magnitude lower than the *i*_corr_ values (0.7389 µA/cm^2^) obtained for CS 1018 in the same period. Therefore, it is shown that even with low mechanical properties, less dense concrete matrix and high permeability, the durability of GC is increased by presenting excellent resistance to corrosion when exposed to 3.5 wt.% Na_2_SO_4_ for more than 364 days, associated with the excellent corrosion performance of AISI 304 SS as reinforcement in concrete exposed to aggressive media.

## Figures and Tables

**Table 1:** Physical properties of the natural coarse aggregate (NCA), natural fine aggregate (NFA) and recycled coarse aggregate (RCA).

Type of Aggregates	Relative Density (Specific Gravity)	Bulk Density (Unit Weight, kg/m^3^)	Absorption (%)	Fineness Modulus	Maximum Aggregate Size (mm)
NCA	2.62	1433	1.73	-	19
NFA	2.24	1695	1.85	2.2	-
RCA	2.20	1367	12.00	-	19

**Table 2:** Proportioning of concrete mixtures in kg for 1 m^3^ of concrete ($f_{c}^{\prime }$ = 22.5 MPa).

Materials	MC (100% CPC)	M50 (50% RCA)	M100 (100% RCA)
		Kg/m^3^	
Cement	315	252	252
Water	205	205	205
SCBA	0	63	63
NCA	917	458.5	0
NFA	914	914	914
RCA	0	458.5	917

**Table 3:** Physical properties of concrete mixtures.

Concrete Mixture	Slump (cm)	Temperature (°C)	Density (kg/m^3^)
MC	10 cm	24	2220
M50	3 cm	19	2187
M100	2 cm	22	2040

**Table 4:** Compressive strength at 14 and 28 days ($f_{c}^{\prime }$ in MPa).

Concrete Mixture	Compressive Strength (MPa)	
	14 Days	28 Days
MC	14.02	19.91
M50	7.71	11.54
M100	6.75	9.66

**Table 5:** Elemental composition (wt.%) of the reinforcements tested, AISI 1018 carbon steel (CS) and austenitic AISI 304 SS.

Material	Element, wt.%									
	C	Si	Mn	P	S	Cr	Ni	Mo	Cu	Fe
AISI 1018	0.20	0.22	0.72	0.02	0.02	0.13	0.06	0.02	0.18	Balance
AISI 304	0.04	0.32	1.75	0.03	0.001	18.20	8.13	0.22	0.21	Balance

**Table 6:** Nomenclature of the reinforced green concrete specimens for electrochemical monitoring.

Mixtures Concrete	Nomenclature of Specimens Exposed DI-Water (Control Medium)		Nomenclature Specimens Exposed to 3.5 wt.% Na_2_SO_4_ Solution (Aggressive Medium)	
MC (Conventional Concrete: 100% NA and 100% CPC)	MC-W-18	MC-W-304	MC-S-18	MC-S-304
M50 (Green Concrete: 50% RCA and 20% SBCA)	M50-W-18	M50-W-304	M50-S-18	M50-S-304
M100 (Green Concrete: 100% RCA and 20% SBCA)	M100-W-18	M100-W-304	M100-S-18	M100-S-304

**Table 7:** The measured half-cell corrosion potential (*E*_corr_) versus a Cu/CuSO_4_ in reinforcement concrete [86].

*E*_corr_ (mV_CSE_)	Corrosion Condition
*E*_corr_ > −200	Low (10% of risk corrosion)
−200 > *E*_corr_ > −350	Intermediate corrosion risk
−350 > *E*_corr_ > −500	High (<90% of risk corrosion)
*E*_corr_ < −500	Severe corrosion

**Table 8:** Level of corrosion in concrete, corrosion current density (*i*_corr_) and the corrosion rate (*v*_corr_) [84].

*i*_corr_ (µA/cm^2^)	*v*_corr_ (mm/d)	Corrosion Level
*i*_corr_ ≤ 0.1	*v*_corr_ ≤ 0.001	Negligible (Passivity)
0.1 < *i*_corr_ < 0.5	0.001 < *v*_corr_ < 0.005	Low Corrosion
0.5 < *i*_corr_ < 1	0.005 < *v*_corr_ < 0.010	Moderate Corrosion
*i*_corr_ > 1	*v*_corr_ > 0.010	High Corrosion
